# A Multistep DNA-Based Methodology for Accurate Authentication of Sturgeon Species

**DOI:** 10.3390/foods11071007

**Published:** 2022-03-29

**Authors:** Andreea Dudu, Maria Samu, Marilena Maereanu, Sergiu Emil Georgescu

**Affiliations:** 1 Department of Biochemistry and Molecular Biology, Faculty of Biology, University of Bucharest, 050095 Bucharest, Romania; andreea.dudu@bio.unibuc.ro (A.D.); mariasamu95@yahoo.com (M.S.); 2 Research Department, SC Danube-Research Consulting, 013172 Isaccea, Romania; marilena.maereanu@gmail.com

**Keywords:** caviar, sturgeon identification, hybrids, nuclear markers, DNA barcoding, molecular diagnostic

## Abstract

The sturgeons (order Acipenseriformes) are caviar producers and some of the most valuable fish species worldwide. Due to different reasons, wild populations are now at the brink of extinction. The high demand for caviar has led to the development of aquaculture for restocking and caviar production. Since the caviar from different species has different prices depending on the quality and attempts of commercial fraud based on species substitution have been found, correct species identification is more than necessary. We report a new multistep methodology for an accurate species identification based on both nuclear and mitochondrial markers. Our test integrates data from the analysis of microsatellites (Afu19, Afu34, Afu39, Afu54, Aox27, AoxD234, AnacC11 and AnacE4), nuclear gene markers (RPS7, vimentin and rhodopsin) and mtDNA barcoding to give a reliable molecular diagnostic for five sturgeon species (*Huso huso, Acipenser stellatus, Acipenser ruthenus, Acipenser gueldenstaedtii* and *Acipenser baerii*). In addition to species identification, our methodology allows the identification of bester, sterbe and best beluga hybrids, but also the identification of hybrids of unknown origin. This methodology has a good potential to contribute to the conservation of highly threatened sturgeon populations and also to the traceability of their products.

## 1. Introduction

Sturgeons (order Acipenseriformes) represent some of the oldest fish species, as they are considered genuine “living fossils” and for this reason they can provide a model for the evolution of vertebrates [1]. For most people, sturgeons are synonymous with caviar, the salted eggs from these species and their hybrids, and one of the most refined food products. Over time, these fish, that are found in the Northern Hemisphere only, have been experienced a drastic decline worldwide, which has been caused by factors such as pollution, dam-building, overfishing and poaching for caviar production. As a consequence, all 27 species of order Acipenseriformes are included on the IUCN Red List, with 17 of these being classified as critically endangered, two endangered and four vulnerable [2]. Moreover, for conservation, since 1998, international trade in all species of sturgeons has been regulated under the Convention on International Trade in Endangered Species of Wild Fauna and Flora (CITES) [3].

To reduce the pressure on wild populations, sturgeon aquaculture has grown steadily in recent decades, mainly for two reasons: to supply the market with caviar and to support restocking programs as it is very well known that the wild populations cannot survive without artificial breeding and restocking [4,5].

In Romanian fish farms, for example, valuable species in terms of producing high-quality caviar (beluga sturgeon—*Huso huso*, stellate sturgeon—*Acipenser stellatus*, Russian sturgeon—*Acipenser gueldenstaedtii*) or high-quality meat (sterlet—*Acipenser ruthenus*) are now reared. In addition to these species, found on the verge of extinction in the Danube River, fish farms rear exotic species, such as the Siberian sturgeon (*Acipenser baerii*), and different interspecific hybrids with high commercial value [6,7]. The phenomenon of interspecific hybridization occurs very rarely among vertebrates because the parental genomes are incompatible and the hybrids formed are sterile [8]. Sturgeons have a complex genome and different species are characterized by different states of ploidy [9,10,11]. The species with ~120 chromosomes are functional diploids, the ones with ~250 chromosomes are tetraploids and the ones with ~370 chromosomes are hexaploid [12]. Despite their complexity, it has been observed, however, that sturgeons can produce interspecific and even intergeneric hybrids that are viable and fertile. Hybridization is more common in aquaculture compared to the natural environment, where it occurs mainly due to the construction of dams or the release of non-native species in a habitat [13,14]. A variety of crosses between hybrids and backcrosses of hybrids with their parental species have been described. These are mainly produced for aquaculture under the consideration that a hybrid has an improved performance compared to the pure species under production conditions. Generally, the hybrids from aquaculture result from species with the same level of ploidy and have improved characteristics compared to the parental species. For example, the bester hybrid (*H. huso* × *A. ruthenus*) has a higher growth rate compared to the paternal species, *A. ruthenus*, and reaches sexual maturity faster and produces higher quality caviar than the maternal species, *H. huso* [15,16].

In this context, sturgeon species identification is critical for several reasons: breeding, restocking, conservation and avoidance of illegal and mislabeled trade of fisheries products. Numerous attempts of fraud have been found. Sometimes, caviar is replaced with that from other, less valuable, sturgeon or from other species of fish and sold in the market as a mislabeled high-value product [17,18,19]. Furthermore, various other products called “caviar” that have no trace of fish eggs but simulate the taste of caviar have been identified in the market [20].

Initially, sturgeon species were identified using morphological or biochemical methods. Both methods presented some drawbacks. Species identification based on morphology has the benefit of being low cost and time efficient, but is possible for intact fish only, reliable for adult individuals only and very difficult to accomplish for hybrids. Moreover, a major disadvantage of this method is that it is not suitable for meat and caviar. Species detection based on biochemical markers (e.g., allozymes) presents major disadvantages if samples are processed. To avoid these difficulties, DNA-based tools have been increasingly used for species detection in the last two decades. Species identification by using DNA markers presents multiple advantages compared with other methods. For example, DNA is thermostable and can be isolated from various tissues (fin, scale, muscle and roe) and even from processed products such as meat and caviar. Moreover, a small amount of sample is necessary for analysis, the results are reproducible and confirmable in different laboratories and the costs are affordable compared with other methods [21,22,23].

For the abovementioned reason, different authentication methods based on molecular markers were proposed. Thus, polymerase chain reaction–restriction fragment length polymorphism (PCR-RFLP), random amplified polymorphic DNA (RAPD), amplified fragment length polymorphism (AFLP), COIBar-RFLP, microsatellite analysis, PCR-specific primers or sequencing were tested for species identification. When compared, the methods are very diverse in terms of efficiency, accuracy, costs and rapidity [5,14,20,21,22,23,24].

Over the time, both mitochondrial and nuclear markers were used for species detection. Mitochondrial DNA (mtDNA) analysis is the main standard of species identification, with the 5′ end of the mitochondrial gene COI being largely employed as a barcode to identify and authenticate animal species. Besides this molecular barcode, markers such as the cytochrome b (cyt b) gene and control region (D-loop) were widely used for sturgeon species identification. Moreover, 25 species of the *Acipenseridae* family have the sequence of the mitochondrial genome deposited in GenBank [25]. The mitochondrial DNA is almost exclusively inherited in a clonal mode from mother to offspring. Thus, this presents a limitation in sturgeon hybrid identification. Here, it is useful only for maternal species detection. It was also highlighted that some mitochondrial markers do not differentiate between the closely related species of the “*gueldenstaedtii* complex” [26,27]. Thus, in recent years the studies on sturgeon species identification have been focusing on bi-parentally inherited nuclear markers with the aim of detecting hybrids. Two types of markers, microsatellites and single nucleotide polymorphisms (SNPs), have been proven to be suitable for pure species and hybrid detection. Microsatellites (or simple sequence repeats—SSRs) are tandem repeats of 1–6 bp with a high level of variability. Due to their great polymorphism they are very useful for the study of genetic differentiation at various levels (individual, population, species) [21]. The continuous development of next generation sequencing (NGS) methods has led to identification of numerous SNPs that can be further investigated regarding their diagnostic strength. Once identified, the so-called “private” SNPs can be used for species discrimination. In sturgeons, this type of approach has led to satisfactory results [28,29,30].

Despite their proven suitability for forensic studies, both microsatellites and SNPs have some drawbacks due to analytical limitations that can lead to misidentification. In the case of microsatellites, different analytical conditions might cause a small amount of allele shifting, so the same individual investigated in different laboratories may have slight differences in allelic size. The validation of SNPs as markers for species identification requires a consistent number of samples from different geographical locations to have a better representation of the genetic variability. For this reason, it is highly recommended that more than one marker is used to ensure a correct species diagnostic.

The current study proposes a multistep methodology based on nuclear and mitochondrial markers for correct species assignation in five sturgeon species (*H. huso*, *A. stellatus*, *A. ruthenus*, *A. gueldenstaedtii* and *A. baerii*) and their hybrids. A panel of eight microsatellites (Afu19, Afu34, Afu39, Afu54, Aox27, AoxD234, AnacC11 and AnacE4) and three nuclear gene markers (RPS7, vimentin and rhodopsin) combined with DNA barcoding of the COI mitochondrial region was proposed for the diagnostic of the previously mentioned sturgeons. Besides the multistep approach for an accurate diagnostic, we propose for the first time the rhodopsin gene as a putative marker for sturgeon species identification.

## 2. Materials and Methods

### 2.1. Schematic Overview of the Experimental Program

**Figure 1:**
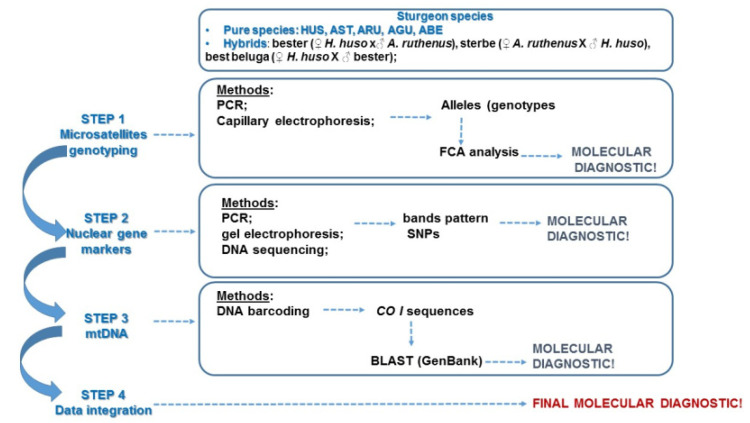
Flow diagram of the experimental procedures used for the set-up of multistep DNA-based method for sturgeon species detection.

An overview of the workflow of this study can be summarized as follows: the sampling included both pure species and aquaculture hybrids, but also two samples of putative hybrids (considered this way based on morphological traits). Following DNA isolation, a multistep analysis was carried out using: (i) nuclear markers including (1) microsatellite genotyping and (2) nuclear genes analyzed in order to give a molecular diagnostic both for pure species and hybrids; (ii) mtDNA analysis by DNA barcoding for species validation or maternal species identification in the case of hybrids. The final diagnostic will integrate the overall set of molecular data (Figure 1).

### 2.2. Sampling and DNA Extraction

A total of 210 fishes from five species and hybrids were included in the study (Table 1).

The biological samples representing fins, meat or eggs were collected from two fish farms from Romania and the Lower Danube River from 2014–2020. Each sample was preserved in 2 mL labeled tubes filled with 96% ethanol and stored at −20 °C prior to DNA extraction. Total genomic DNA was extracted from the collected samples using a phenol–chloroform–isoamylic alcohol protocol [31] or the DNeasy tissue kit (Qiagen, Hilden, Germany) following the manufacturer’s instructions with some modifications. The quality and quantity of the DNA were assessed by using a NanoDrop 8000 (Thermo Fisher Scientific, Waltham, MA, USA). DNA samples were stored at −20 °C until further analysis.

### 2.3. Microsatellite Genotyping and Data Analysis

Microsatellite analysis was performed using a panel of eight loci (Afu19, Afu34, Afu39, Afu54, Aox27, AoxD234, AnacC11 and AnacE4) previously described for other sturgeon species [32,33,34,35]. These eight loci were selected after preliminary testing of numerous loci because they showed highly reproducible amplification, high polymorphism, conformity and the absence of null alleles in analyzed species [36]. Amplifications were performed on a GenAmp PCR System 9700 (Perkin Elmer, Applied Biosystems Division, Foster City, CA, USA) thermocycler with a 25 μL reaction volume containing 30–50 ng of DNA template, 1*×* PCR Buffer, 1.5 mM of MgCl_2_, 0.4 μM of each primer, 0.8 mM of each dNTP and 0.5U of AmpliTaq Gold Polymerase (Perkin Elmer, Applied Biosystems Division, Foster City, CA, USA). Cycling conditions were as follows: denaturation at 95 °C for 5 min; then 30 cycles of 30 s at 95 °C, 30–45 s at specific annealing temperature and 1 min at 72 °C and a final extension at 72 °C for 5 min (Table 2).

The PCR products were mixed with HiDi formamide and molecular size standard GeneScan−500 LIZ to determine the sizes of amplified DNA fragments. Electrophoretic separation of amplification products was performed using an ABI 3130 Genetic Analyzer (Perkin Elmer, Applied Biosystems Division, Foster City, CA, USA) system of capillary electrophoresis and the results were visualized with the GeneScan 3.1.2 software (Perkin Elmer, Applied Biosystems Division, Foster City, CA, USA). The alleles were scored with Genotyper 2.5.2 software (Perkin Elmer, Applied Biosystems Division, Foster City, CA, USA).

Some loci displayed tetrasomic banding patterns for Russian sturgeon (AGU) and Siberian sturgeon (ABE), which are considered tetraploid species (with an octaploid ancestor) that are still in the process of diploidization. Thus, two, four or more than four alleles can be scored for each locus. Since the aim of the study was the correct assignation of the species, the true genotypes in this case were not determined. Moreover, for data analysis, the polyploid genotypes were “artificially” transformed into several diploid ones according to the method described in Dudu et al., 2012 [36].

Each individual was probabilistically tested for belonging to each of the presumptive species using GENETIX software (Université de Montpellier II, Montpellier, France) [37]. A factorial correspondence analysis (FCA) was carried out in order to investigate the relationships among individuals. This type of analysis can explain a maximal amount of genetic variation using a minimal number of factors and it can provide the means for visualizing the genetic relationships between populations/species. Allelic frequencies of pure species samples were calculated with the abovementioned software to investigate the occurrence of species-specific alleles.

### 2.4. Nuclear Gene Marker Analysis

Six published nuclear primers used previously for species identification in fish species were tested on five individuals from each pure/hybrid species in order to verify their cross-amplification and suitability for accurate species assignment in sturgeons (Table 3).

To check the cross amplification of primers in the analyzed species, a temperature gradient PCR was performed. The amplification program was: denaturation at 95 °C for 2 min, 35 cycles of: denaturation at 95 °C for 30 s, annealing at gradient temperature 50–60 °C for 30 s and extension at 72 °C for one minute, with final extension at 72 °C for 10 min. The PCR reactions were carried out in a final volume of 25 μL with 1× PCR Buffer, 1.5 mM of MgCl_2_, 200 μM of each nucleotide, 0.4 μM of each primer, 0.5 units of AmpliTaq DNA polymerase (Perkin Elmer, Applied Biosystems Division, Foster City, CA, USA), nuclease-free water and 50 ng of DNA template. The PCR products were evaluated by agarose gel electrophoresis. No amplification was observed for 5S rRNA and parvalbumin in any of the sturgeon pure species/hybrids, so these markers were excluded for identification of pure sturgeon species and hybrids.

The final set of markers included: (i) the first intron RP1 of the RPS7 gene that was amplified by universal primers (S7RPEx F and S7RPEx1R) and by specific primers for sterlet and Siberian sturgeon (Rut_Bae F and RP1_LocusA R); (ii) a fragment of the vimentin gene that was amplified by primers designed to amplify alleles present in *A. baerii* and (iii) the rhodopsin gene. For these markers, the amplification was carried out for all the individuals.

Following amplification, the PCR products were checked by 2% agarose gel electrophoresis.

For rhodopsin, the amplification products were purified with Wizard SV Gel and PCR Clean-Up System (Promega, Madison, WI, USA). Separate forward and reverse strand sequencing amplification was carried out using a BigDye Terminator v3.1 Kit (Perkin Elmer, Applied Biosystems Division, Foster City, CA, USA). The resulting products were purified using the BigDye XTerminator Purification Kit (Perkin Elmer, Applied Biosystems Division, Foster City, CA, USA), followed by sequencing on the ABI Prism 3130 DNA Genetic Analyzer (Perkin Elmer, Applied Biosystems Division, Foster City, CA, USA) automatic platform. Forward and reverse sequences were analyzed using BioEdit [42] and analyzed and authenticated with the web Basic Local Alignment Search Tool (BLAST) [43] on GenBank and the multiple alignment was carried out with Clustal X [44] using the obtained rhodopsin sequences in order to identify species-specific SNPs.

### 2.5. DNA Barcoding

This method was used to confirm the pure species detected in previous steps and the origin of the maternal genitor in the case of hybrids. The amplification of a 5′ fragment of the cytochrome oxidase subunit 1 (COI) gene was carried out using the following primers: SturF: 5′GAAGGGGACTTTAACCTCTG3′/Stur R: 5′TAGGCCCGTGTGTCTACGTCC3′, which were designed using the Primer3 software [45]. The PCR amplifications were performed in a 25 µL final reaction volume containing: 50 ng of DNA template, 1× PCR buffer (Perkin Elmer, Applied Biosystems Division, Foster City, CA, USA), 1.5 mM MgCl_2_ (Perkin Elmer, Applied Biosystems Division, Foster City, CA, USA), 0.4 mM of each dNTP (Perkin Elmer, Applied Biosystems Division, Foster City, CA, USA), 0.4 µM of F primer, 0.4 µM of R primer, 1 U of AmpliTaq Gold Polymerase (Perkin Elmer, Applied Biosystems Division, Foster City, CA, USA). The PCR reaction conditions were: 95 °C for 10 min, 35 cycles of 95 °C for 30 s, 54 °C for 30 s and 72 °C for 60 s, followed by a final extension of 72 °C for 10 min and the amplifications were performed on a Verity Thermal Cycler (Applied Biosystems). The resulting PCR products were assessed for quality by agarose electrophoresis, and purified using Wizard SV Gel and PCR Clean-Up System (Promega). Separate forward and reverse strand sequencing amplification was carried out using a BigDye Terminator v3.1 Kit (Perkin Elmer, Applied Biosystems Division, Foster City, CA, USA). The resulting products were purified using the BigDye XTerminator Purification Kit (Perkin Elmer, Applied Biosystems Division, Foster City, CA, USA), followed by sequencing on the ABI Prism 3130 DNA Genetic Analyzer (Perkin Elmer, Applied Biosystems Division, Foster City, CA, USA) automated platform.

Sequences were edited with BioEdit Sequence Alignment Editor and screened using the BLAST algorithm to identify the nearest matching sequences in the GenBank database.

## 3. Results

### 3.1. Step 1: Microsatellite Genotyping

The most polymorphic locus is Afu54 with 26 alleles, while Afu34 exhibited the lowest polymorphism with eight alleles in the analyzed data set. The total number of alleles per species ranged from one in *A. stellatus* and *H. huso* to 10 in *A. gueldenstaedtii* (ST1). The allelic frequencies were calculated to identify potential private alleles with a value of diagnostic alleles. The allelic frequencies were distributed from 0.01 to 1. No diagnostic alleles were highlighted, except one for Afu39 in *A. stellatus* that was already reported [46].

A multidimensional analysis was performed as follows:

*(i)*  *On genotype data set of pure species*.

**Figure 2:**
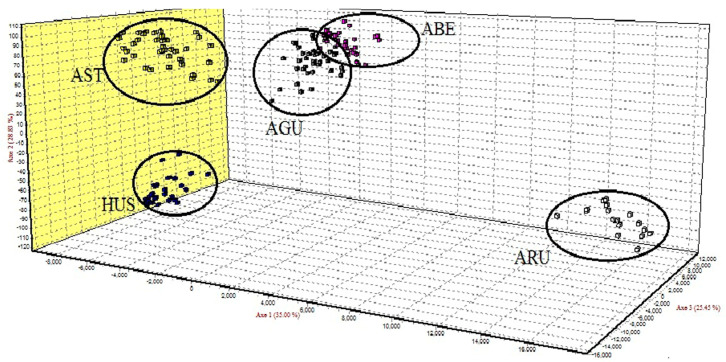
Factorial correspondence analysis (FCA) based on microsatellite data in five sturgeon species. HUS—*H. huso*; AST—*A. stellatus*; ARU—*A. ruthenus*; AGU—*A. gueldenstaedtii*; ABE—*A. baerii*.

The FCA has emphasized the identification of three pure species based on microsatellite analysis. Three clusters, corresponding to stellate sturgeon (STE), beluga sturgeon (HUS) and sterlet sturgeon (RUT), were identified. Russian sturgeon (AGU) and Siberian sturgeon (ABE) individuals clustered together, as for these two species the diagnostic based on microsatellite analysis was totally ambiguous (Figure 2).

*(ii)*  *On a data set comprising the genotypes of hybrids bester, sterbe and best beluga together with their genitor species, A. ruthenus and H. huso.*

**Figure 3:**
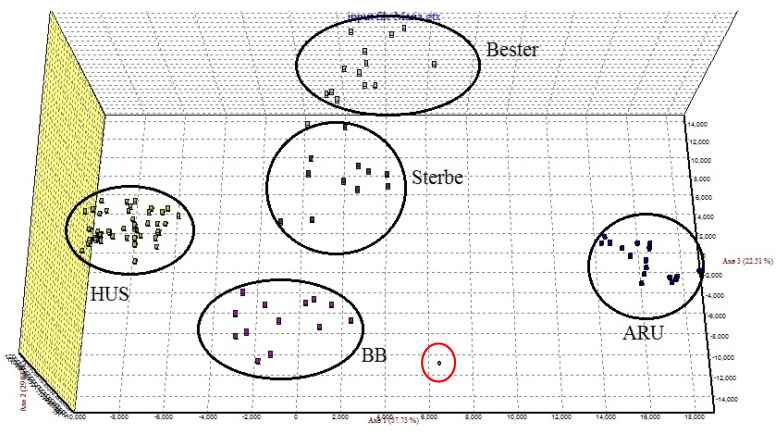
Factorial correspondence analysis (FCA) based on microsatellite data in two sturgeon species and their interspecies hybrids. HUS—*H. huso*; ARU—*A. ruthenus*; BB—best beluga.

Here, five clusters were inferred. The genitor species are completely separated, while the hybrids are placed in specific clusters distributed between the two corresponding to the genitor species. Some individuals of bester and sterbe hybrids fall out of the assigned cluster and this led to uncertain diagnostics. The best beluga hybrid cluster is differentiated from both the genitors and from the other two hybrids (Figure 3).

*(iii)*  *On the data set that consists in genotypes of pure species and two putative hybrids captured in the Danube River.*

**Figure 4:**
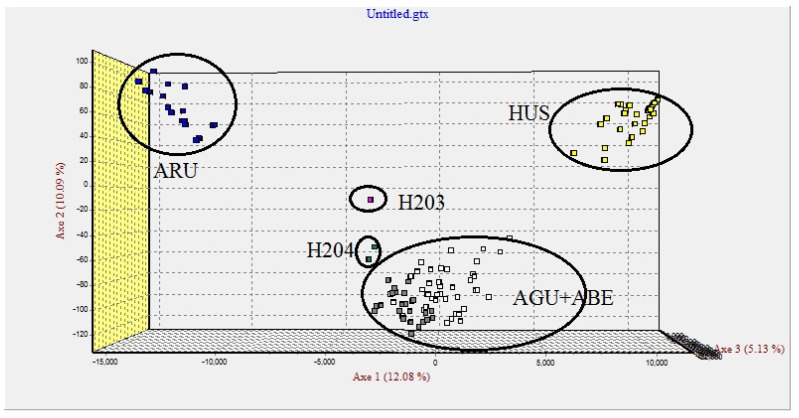
Factorial correspondence analysis (FCA) based on microsatellite data in four sturgeon species and two putative hybrids (H203 and H204). HUS—*H. huso*; ARU—*A. ruthenus*; AGU—*A. gueldenstaedtii*; ABE—*A. baerii*.

DNA samples were isolated from two fish captured in the Lower Danube and having peculiar morphometric features that led to the hypothesis that these are in fact hybrids. The putative hybrids (sample code: H203 and H204) were analyzed together with individuals from the five species included in the study. First, the FCA highlighted that *A. stellatus* clustered entirely separately and has no contribution as a genitor species for the hybrids H203 and H204. To simplify the analysis, the genotypes of AST were removed from the next FCA test. Consequently, the multidimensional analysis showed completely separated clusters for *H. huso, A. ruthenus* and *A. gueldenstaedtii* + *A. baerii*. The putative hybrids were placed in the interior of the triangle delimited by these three clusters: H203 is in the middle of the triangle, while H204 is closer to the *A. gueldenstaedtii* + *A. baerii* cluster, highlighting the possibility of one of these two species being a genitor species (Figure 4).

Moreover, this hypothesis is sustained by the fact that H204 has a polypoid genotype, showing more than two alleles for three loci.

### 3.2. Step 2: Nuclear Gene Analysis

Four loci (S7RPEx1, RP1, vimentin, rhodopsin) were amplified with universal or species-specific primers. The first objective regarding these nuclear markers was to determine whether a species-specific band pattern resulted following the electrophoresis. This would simplify the identification of sturgeon species and of their interspecies hybrids since a tool based on simple PCR and gel electrophoresis is fast, inexpensive and reproducible.

*(i)*  *RP1*.

**Figure 5:**
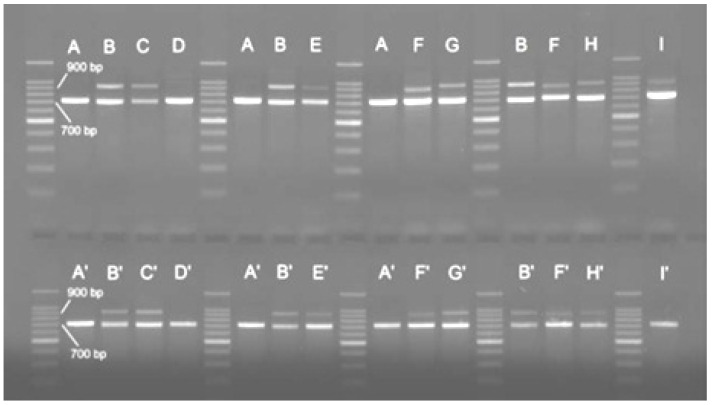
Band patterns given by amplifying the RP1 locus with universal primers. A, A’—*H. huso*; B, B’—*A. ruthenus*; C, C’—bester; D, D’—best beluga; E, E’—sterbe; F, F’—*A. gueldenstaedtii*; G, G’—H203; H, H’—H204; I, I’—*A. stellatus*. The band size was estimated by using a 100 bp DNA Ladder (Promega).

The first intron RP1 of the ribosomal protein RPS7 was amplified by using universal primers. After performing an agarose gel electrophoresis, two bands of approximatively 700 bp and 900 bp, respectively, were inferred (Figure 5).

This band pattern was observed for all individuals, except those of *H. huso*. For this species, all individuals exclusively presented the band of 700 bp. A similar situation was found in best beluga, the hybrid of *H. huso* with bester, for which the 900 bp was not observed for nine of the twelve analyzed individuals.

**Figure 6:**
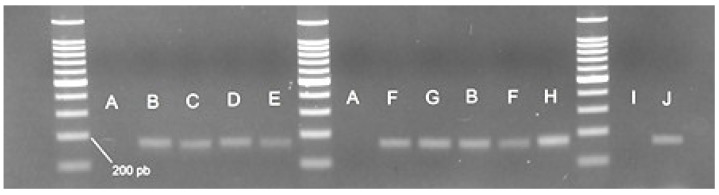
Band patterns given by amplifying the locus A of RP1 with species-specific primers Rut_Bae F/RP1_LocusA R. A—*H. huso*; B—*A. ruthenus*; C—bester; D—best beluga; E—sterbe; F—*A. gueldenstaedtii*; G—H203; H—H204; I—*A. stellatus*; J—*A. baerii*. The band size was estimated by using a 100 bp DNA Ladder (Promega).

Locus A of RP1 was also amplified using species-specific primers for *A. ruthenus* and *A. baerii*, resulting in a 169 bp fragment. Following electrophoresis, we observed that the amplification was positive for sterlet (*A. ruthenus*) and Siberian sturgeon (*A. baerii*), as expected. Moreover, these primers give positive amplifications for non-target species such as *A. gueldenstaedtii.* A 169 bp band was also observed in AGU in seven of the 15 individuals. The same amplification product was observed in interspecies hybrids of *H. huso* and *A. ruthenus*, namely, bester, sterbe and best beluga. Positive amplifications were also demonstrated for H203 and H204. No amplification was observed in any individuals of *H. huso* and *A. stellatus* (Figure 6).

For five individuals of *A. ruthenus* and, respectively, *A. baerii*, the fragment was sequenced and the obtained sequences were aligned for SNP detection (Supplementary Figure S1). The alignment of the 169 bp sequences showed that there are no intraspecies polymorphisms. Instead, two interspecies SNPs were identified in *A. baerii* and *A. ruthenus* (Table 4).

The sequences were compared with similar ones from GenBank. The BLAST analysis for ARU sequences showed 100% identity with the sequence KF771098 representing a partial sequence of the ribosomal protein S7 (rpS7) gene in *A. ruthenus*. The sequences of ABE exhibited 100% similarity with the sequence OV737687 and 99.41% similarity with the sequence OV754675, representing genome assemblies of chromosome 6 in *A. ruthenus*. A 98.82% similarity was observed for sequences of the rpS7 gene in *A. baerii* (KF771099) and in *A. ruthenus* (KF771098).

*(ii)*  *Vimentin (vim)*.

The vimentin locus was amplified with Bae154B7F/Bae154B7R reported in the literature to specifically amplify alleles in *A. baerii*, but rarely found in *A. gueldenstaedtii*, to obtain a 373 bp fragment.

**Figure 7:**
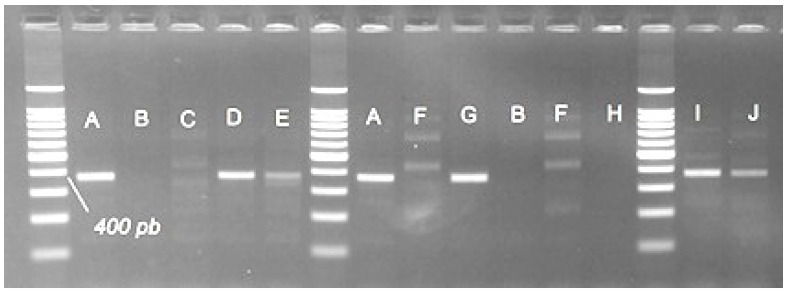
Band patterns given by amplifying the locus vimentin with species-specific primers Bae154B7F/Bae154B7R. A—*H. huso*; B—*A. ruthenus*; C—bester; D—best beluga; E—sterbe; F—*A. gueldenstaedtii*; G—H203; H—H204; I—*A. stellatus*; J—*A. baerii*. The band size was estimated by using a 100 bp DNA Ladder (Promega).

The electrophoretic profile revealed positive amplification for *A. baerii* and *H. huso* and partially for *A. stellatus* (32 of 40 individuals). The 373 bp fragment was also present in the hybrids sterbe, best beluga and H203. No amplification was observed in the individuals of *A. ruthenus*, bester and hybrid H204. The band of 373 bp is absent in *A. gueldenstaedtii* where a different band profile was shown for all individuals. The amplification of this marker by the abovementioned primers resulted in an amplification of a maximum of four alleles around 200, 450, 700 and 900 bp in AGU individuals (Figure 7).

For five individuals of *A. baerii*, *A. stellatus* and *H. huso*, the 373 bp fragment was sequenced and the obtained sequences were aligned for SNP detection (Supplementary Figure S2). The alignment of sequences revealed that the sequences are identical, except the individuals of *H. huso* who presented a SNP (Table 4). For the hybrids sterbe, best beluga and H203, the sequences were identical to those of pure species.

The sequences were compared with similar ones from GenBank. All presented ~98% similarity with a partial sequence of the vimentin gene from *A. baerii* (AJ493226).

*(iii)*  *Rhodopsin (Rh).*

**Figure 8:**
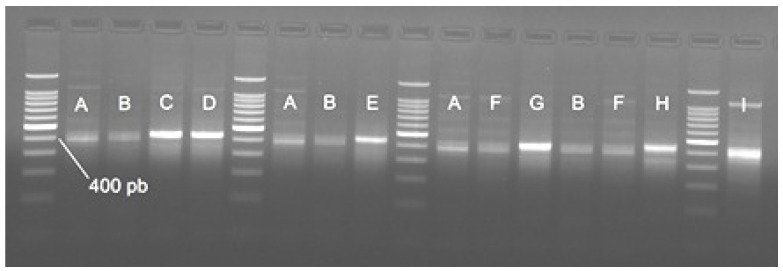
Band patterns given by amplifying the locus rhodopsin with universal primers. A—*H. huso*; B—*A. ruthenus*; C—bester; D—best beluga; E—sterbe; F—*A. gueldenstaedtii*; G—H 203; H—H 204; I—*A. stellatus*. The band size was estimated by using a 100bp DNA Ladder (Promega).

The amplification of this marker with universal primers followed by electrophoresis showed a multiband pattern in the pure species and hybrids, with a 400 bp band present in all cases (Figure 8).

The band of about 400 bp present in the analyzed species was sequenced for five individuals of *A. stellatus, A. ruthenus*, *A. gueldenstaedtii* and *H. huso*. The alignment of 385 bp highlighted the presence of SNPs with possible diagnostic value (Supplementary Figure S3, Table 4). The BLAST analysis showed a similarity of ~98% with rhodopsin sequences from *A. baerii* (LC438462) and *A. ruthenus* (XM_034023211).

### 3.3. Step 3: mtDNA Analysis

The designed primers STUR F/STUR R amplify a 976 bp region of the COI gene. The sequences were truncated to accommodate a 5′ COI gene that is used as a standard barcode in animal species.

The 654 bp fragment was compared with sequences from GenBank and the results are summarized in Table 5.

### 3.4. Step 4: Data Integration

All the data resulting from microsatellites, nuclear gene markers and mtDNA analysis were correlated in order to give an accurate diagnostic of both pure species and hybrids. The markers used in each step of analysis were investigated to evaluate their power of discrimination for each of the pure species and hybrids that we tested (Table 6).

## 4. Discussion

We proposed this multistep DNA-based method for accurate species discrimination in sturgeons in the light of the tremendous efforts that were made in the last decade to identify, test and validate DNA markers for sturgeon identification. The method is based on analyzing both nuclear and mitochondrial markers and integrates the data for the final diagnostic. Each of the proposed markers was also examined for its power in species and hybrid detection on its own. The approach proposed here is based on initially analyzing the nuclear markers that, due to their inheritance pattern, can distinguish between pure species and hybrids. This aspect is essential for various reasons, but mainly because, nowadays, a significant part of aquaculture caviar production is attributed to hybrids.

The microsatellites that we analyzed in the study showed a good power to correctly identify three of five pure species. Thus, the stellate sturgeon (*A. stellatus*), beluga sturgeon (*H. huso*) and sterlet sturgeon (*A. ruthenus*) were clearly differentiated by the genotype data resulting from a set of eight microsatellites. These markers were unable to differentiate between the two tetraploid species included in the so-called “*gueldenstaedtii* complex”, *A. gueldenstaedtii* and *A. baerii*, and this is a drawback considering their great commercial value. Here, the selection of new microsatellite loci to complete the current panel can be useful. This set of eight microsatellites can differentiate between the four species native in the Danube River (*A. stellatus*, *A. gueldenstaedtii*, *A. ruthenus* and *H. huso*), but not between *A. gueldenstaedtii* and *A. baerii*.

This multilocus approach based on microsatellite analysis has a good power to discriminate between the pure species and hybrids. When an FCA test was run on the genotype data resulting from the analysis of bester, sterbe and best beluga hybrids and their genitor species *H. huso* and *A. ruthenus*, the hybrids were differentiated from their genitor species. Moreover, the microsatellites can distinguish best beluga from the other two hybrids. The analyzed individuals of this hybrid produced in aquaculture by back crossing the bester hybrid (♀ *H. huso ×* ♂ *A. ruthenus*) with one of its genitor species clustered separately from bester and sterbe hybrids. Concerning these two hybrids, unfortunately, the microsatellites do not differentiate between these two F1 hybrids of *H. huso* and *A. ruthenus*.

For the hybrids H203 and H204, the analysis of microsatellites allowed us to exclude *A. stellatus* as a possible genitor species. The spatial distribution of the H203 hybrid based on the FCA in the middle of a triangle delimitated by the clusters of *A. ruthenus*, *H. huso* and *A. gueldenstaedtii*/*A. baerii* is a clue that any of these species could be involved in the hybrid formation.

Since is already known that microsatellites are useful to detect the ploidy level [9], they can also be good markers for identifying hybrids resulting from genitors of different ploidy levels. For example, a cross between a disomic and a tetrasomic species leads to trisomic hybrids. This is the case of the H204 hybrid in which for the loci Aox27, AoxD234 and AnacC11, three alleles were identified. This indicates as a possible genitor one of the tetraploid species *A. gueldenstaedtii*/*A. baerii*. The FCA indicates a cross between *A. gueldenstaedtii*/*A. baerii* and *H. huso* or *A. ruthenus*.

The second step of the analysis was the analysis of nuclear gene markers that together with microsatellites can discriminate between pure species and hybrids. The nuclear gene markers were selected from already published ones, mainly for two reasons: (i) to differentiate between polyploid species *A. gueldenstaedtii* and *A. baerii* that were not identifiable by using the microsatellites; (ii) to specifically identify *A. ruthenus* and to differentiate it from *H. huso*, since these two species were the genitors of the caviar producer hybrids bester, sterbe and best beluga.

The analysis of the first intron of the ribosomal protein S7 gene by universal primers identified two fragments of 700 bp and 900 bp, respectively, in all species, except *H. huso*. For beluga sturgeon, the 900 bp band was not detected. The *H. huso* individuals analyzed come from two aquaculture strains that resulted from crossing a limited number of breeders from the Lower Danube so are characterized by low genetic variability. This result is different from the one reported in the literature [16] and from what we expected. If the band pattern is confirmed for a higher number of individuals, this nuclear marker might be useful in detecting the beluga sturgeon from the Lower Danube.

The analysis of the vimentin locus certified the differentiation between *A. gueldenstaedtii* and *A. baerii*, since the band of 373 bp is present only in *A. baerii* and it is absent in *A. gueldenstaedtii*, in which a different band pattern was inferred. The primers that we used in this study designed to specifically amplify *A. baerii* only also amplified *A. gueldenstaedtii* from the Caspian Sea, but not *A. gueldenstaedtii* from the Azov Sea [16]. Our study proves the suitability of this locus to also discriminate between *A. baerii* and *A. gueldenstaedtii* originating in the Lower Danube River.

The amplification of locus A of RPS7 (RP1) with specific primers for *A. ruthenus* and *A. baerii* differentiated *A. ruthenus* from *H. huso*, but did not differentiate the hybrids of these two species. The vimentin locus differentiates *H. huso* from *A. ruthenus* and the bester hybrid from sterbe and best beluga. The panel of microsatellites and nuclear gene markers also allowed differentiation between the pure species and the aquaculture hybrids.

Concerning the hybrids of unknown origin, H203 and H204, that were captured in the Lower Danube, the nuclear gene analysis allowed us, step by step, to identify their genitor species. For H203, the positive amplification of RP1 suggested as a possible genitor *A.* *ruthenus, H. huso* and *A. gueldenstaedtii* (similar to microsatellites). The vimentin locus excludes *A. gueldenstaedtii* as a possible genitor, so for H203 the genitor species are *H. huso* and *A. ruthenus*. The vimentin locus analysis was also relevant in detecting the genitors of the H204 hybrid. The absence of amplification excluded *H. huso* and *A. baerii* and confirmed *A. ruthenus* and *A. gueldenstaedtii* as genitor species.

We tested for the first time rhodopsin as a putative marker for sturgeon identification. The band pattern was ambiguous and did not differentiate between the analyzed species and hybrids. The isolation of a 400 bp band followed by sequencing in the four species native to the Lower Danube (*A. stellatus, A. ruthenus, A. gueldenstaedtii* and *H. huso*) highlighted the presence of two SNPs that if confirmed in a higher number of individuals will specifically identify *A. stellatus* and *H. huso*, respectively. The validation of these SNPs for species identification assumes the analysis of samples from different geographical sites as in this manner we have a better image of the genetic variability of each species. At this point, the identified SNPs are useful to detect stellate sturgeon and beluga sturgeon from the Lower Danube River.

The analysis of mtDNA in the third step showed that this marker is useful to validate the diagnostics based on nuclear markers in the case of pure species and to detect the maternal genitor in the case of hybrids. The DNA barcoding method based on COI sequencing, although used for species detection on a molecular basis in a great variety of animal species, has serious limitations for sturgeons. The BLAST analysis was not conclusive for three of the five pure species included in the study. Thus, the barcode sequences for *A. stellatus* have a 100% identity with a sequence from *A. nudiventris* from GenBank. As the ship sturgeon (*A. nudiventris*) is extinct in the Lower Danube and is not raised in aquaculture, we can exclude this diagnostic.

The identification based on DNA barcoding is even more complicated for the species of the “*gueldenstaedtii* complex”. Thus, the BLAST revealed 100% similarity for our sequences of *A. gueldenstaedtii* with sequences from GenBank coming from the same species, but also for *A. naccarii* and *A. persicus*, two species also included in the “*gueldenstaedtii* complex”. Similarly, *A. baerii* was also identified as *A. gueldenstaedtii*, *A. persicus* and two hybrid species. We can conclude that the DNA barcoding cannot discriminate between species of the “*gueldenstaedtii* complex” and is not recommendable alone for species identification in this group.

The analysis of mtDNA was useful to detect the maternal species for H203 and H204 hybrids and to establish the final diagnostic for these two hybrids of unknown origin as follows: H203 = ♀ HUS *×* ♂ ARU (bester) and H204 = ♀ ARU *×* ♂ AGU.

Since DNA barcoding unquestionably can establish a diagnostic to a genus level in sturgeons, it is suitable to identify the so-called caviar that is produced from other fishes than sturgeon.

The diagnostic value for each of the proposed markers has been established. The pure species/hybrids can be detected by a specific combination of markers as follows:

(i) *A. stellatus*—microsatellites supplemented with RP1 and mtDNA;
(ii) *H. huso*—microsatellites supplemented with S7RPEx1, RP1 and mtDNA;
(iii) *A. ruthenus*—microsatellites supplemented with vimentin and mtDNA;
(iv) *A. gueldenstaedtii*—vimentin + mtDNA;
(v) *A. baerii* –vimentin + mtDNA;
(vi) bester *(H. huso × A. ruthenus)*—microsatellites+ vimentin + mtDNA;
(vii) sterbe *(A. ruthenus × H. huso*)—microsatellites + mtDNA;
(viii) best beluga (*H. huso ×* bester)—microsatellites + mtDNA;
(ix) H203—microsatellites+ RP1 + vimentin + mtDNA;
(x) H204—microsatellites + RP1+ vimentin+ mtDNA.

## 5. Conclusions

DNA-based methods are considered to be the most appropriate tools to identify fish species. However, selecting a method can be very challenging for a group of fish with complex genomes and frequent hybridization such as the sturgeons, although it is extremely important for regulating trade of high-value sturgeon products such as caviar. The NGS studies have suggested a new approach for species identification. New nuclear markers with diagnostic value have been isolated by NGS and then analyzed by PCR with SNP-specific primers. Although this type of method is simple, fast and not expensive, it does not exclude amplification in non-target species and thus a single-locus approach can be risky and lead to an imprecise identification.

Our study demonstrated that the selected microsatellite set is useful to differentiate between some pure species and hybrids, but it is preferable to supplement the analysis with nuclear gene markers and/or mtDNA, when there are ambiguities. For tetraploid species and for hybrids, a combination of microsatellites, nuclear gene markers and mtDNA seems to be imperative for an accurate diagnostic. Our methodology has some limitations because it involves the analysis of multiple markers which can be time consuming. For this reason, our future studies will be directed towards finding and validating a set of nuclear gene markers that will allow the detection of species by PCR. A first step has been shown by this study that proposes the rhodopsin gene as a putative marker for sturgeon species detection. A good set of markers detected by a fast method can replace the current standard analysis based on mtDNA of caviar samples, which is proven to have serious limitations. At this time, our multistep methodology based on both nuclear and mitochondrial marker analysis can accurately detect pure species and hybrids, proving it to be a reliable tool to combat the commercial fraud based on caviar substitution. In addition, this methodology could have a consistent contribution to the management and conservation of this group of fish.

## Figures and Tables

**Table 1:** List of sturgeon (*Acipenseridae* family) species included in the study.

	Common Name	Scientific Name/Genitor Species (in Case of Hybrids)	Abbreviation	No of Individuals	Origin	Sample Type
Pure species	Beluga sturgeon	*H. huso*	HUS	58	Aquaculture	Fin, eggs
	Stellate sturgeon	*A. stellatus*	STE	40	Aquaculture	Fin, eggs
	Russian sturgeon	*A. gueldenstaedtii*	AGU	15	Aquaculture	Fin, eggs
	Siberian sturgeon	*A. baerii*	ABE	15	Aquaculture	Fin
	Sterlet	*A. ruthenus*	RUT	40	Danube River	Fin, meat
Hybrids	Bester	*H. huso* × *A. ruthenus*	−	18	Aquaculture	Fin, eggs
	Sterbe	*A. ruthenus* × *H. huso*	−	10	Aquaculture	Fin
	Best beluga	Bester × *H. huso*	−	12	Aquaculture	Fin, eggs
	Unknown	−	−	2	Danube River	Fin

**Table 2:** Microsatellites analyzed in the study.

Locus	Primer Sequence from 5′ to 3′	Annealing Temperature (°C)	Annealing Time (s)	Reference
Afu19	F: * CATCTTAGCCGTCTGTGGTAC R: CAGGTCCCTAATACAATGGC	55	30	[32]
Afu34	F: * TACATACCTTCTGCAACG R: GATCCCTTCTGTTATCAAC	55	30	[32]
Afu54	F: * CTCTAGTCTTTGTTGATTACAG R: CAAAGGACTTGAAACTAGG	55	30	[32]
Afu39	F: * TTCTGAAGTTCACACATTG R: ATGGAGCATTATTGGAAGG	55	30	[32]
Aox27	F: * AATAACAATAACGGCAGAACCT R: TGTGTTGCTCAAGACAGTATGA	60	45	[33]
AoxD234	F: * AACTGGCTTTGTGATTGATCC R: TGAAGCAAAGGGTATTATTTGAG	52	30	[34]
AnacC11	F: * AAATTTCCATTGGGGTGT R: CTTCGTTTTGAGAACCCG	50	45	[35]
Anac E4	F: * TCAGCTACAGGGTTCTGGG R: GTTGTTACTCATTGGAACTC	55	45	[35]

* PCRs were carried out using fluorescent labeled forward primers.

**Table 3:** Nuclear gene loci analyzed in the study.

Locus	Primer Name/Sequence from 5′ to 3′	Annealing Temperature (°C)	Reference
S7RPEx1	S7RPEx1 F: TGGCCTCTTCCTTGGCCGTC S7RPEx1 R: AACTCGTCTGGCTTTTCGCC	49	[38]
RP1	Rut_Bae F: TTACATTAATTACCTGTGTTAAGATAG RP1_Locus A R: ATCCAAGTACAAGCTTGAACA	49	[16]
Vimentin	Bae154B7F: TCCAGGGTTTCCTACACCAGCCAAT Bae154B7R: CCACCCTCGCTTTTCGTTGGTTTG	59	[16]
Rhodopsin	Rh1F: GTYACCMTBGARCACAAGGAARTC Rh4R: TCRAYYCCRCAYGAGCAYTGCAT	53	[39]
5S rRNA	5S rRNA F: TACGCCCGATCTCGTCCGATC 5S rRNA R: CAGGCTGGTATGGCCGTAAGC	No amplification	[40]
Parvalbumin	Parv F: CAGGACAAGAGTGGCTTCAT Parv R: GAAGTTCTGCAGGAACAGCTT	No amplification	[41]

**Table 4:** Single nucleotide polymorphisms (SNPs) identified in the nuclear markers.

Marker	Species	Nucleotide Position		Marker	Species	Nucleotide Position	Marker	Species	Nucleotide Position			
RP1		**50**	**147**	Vim		60	Rh		13	**262**	**317**	374
	*ARU*	A	G		*AST*	C		*AGU*	G	G	G	G
	*ABE*	T	T		*ABE*	C		*ARU*	A	G	G	A
					*HUS*	C/A		*AST*	G	A	G	A
								*HUS*	A	G	C	G

SNPs in bold, if confirmed in a higher number of samples, have the potential for species identification. The numbers associated with each SNP refer to nucleotide position in the alignment for each nuclear marker.

**Table 5:** List of GenBank screening results generated by BLAST.

Category	Species	Species Matched by BLAST	Matched Accession Number	% Identity
Pure species	*A. stellatus*	*A. stellatus*	JQ623906	100%
		*A. nudiventris*	KC500105	100%
	*A. ruthenus*	*A. ruthenus*	MG648396	100%
			MG648371	100%
			MG648364	100%
			OV754669	100%
			LR824036	100%
	*A. gueldenstaedtii*	*A. gueldenstaedtii*	KJ789859	100%
			FJ392605	100%
		*A. naccarii*	MK078265	100%
		*A. persicus*	MW713795	100%
			MK213065	100%
		*A. sinensis*	EU719645	100%
		*A. gueldenstaedtii* × *A. baerii*	KJ321189	100%
	*A. baerii*	*A. baerii*	KP833617	100%
			MW856904	100%
			MW856903	100%
		*A. gueldenstaedtii*	MT410937	100%
			KM286425	
		*A. persicus*	FJ809722	100%
		*A. gueldenstaedtii* × *A. baerii*	OM049247	100%
		*A. baerii* × *A. schrenckii*	KC578843	100%
	*H. huso*	*H. huso*	AY442351	100%
Hybrids	Bester	*H. huso*	AY442351	100%
	Sterbe	*A. ruthenus*	MG648396	100%
			MG648371	100%
			MG648364	100%
			OV754669	100%
			LR824036	100%
	Best beluga	*H. huso*	AY442351	100%
	H203	*H. huso*	AY442351	100%
	H204	*A. ruthenus*	OV754669	100%
			LR824036	100%

**Table 6:** Data integration for all DNA markers. Each marker is evaluated for its accuracy in pure species and hybrid identification. D—diagnostics value; BP—band pattern; NS—not specific (band pattern); ?—ambiguous (needs further investigation).

		Microsatellites	Nuclear Gene Markers												DNA Barcoding
		D	S7RPEx1			RP1			Vimentin			Rhodopsin			D
			BP	SNPs	D	BP	SNPs	D	BP	SNPs	D	BP	SNPs	D	
Pure species	*A. stellatus*	YES	700/900	−	NO	−	−	YES	373	−	NO	NS	+	?	YES
	*H. huso*	YES	700/700	−	YES	−	−	YES	373	−	NO	NS	+	?	YES
	*A. ruthenus*	YES	700/900	−	NO	169	+	?	−	−	YES	NS	+	?	YES
	*A. gueldenstaedtii*	NO	700/900	−	NO	169	+	?	200/450/700/900	−	YES	NS	+	?	YES
	*A. baerii*	NO	700/900	−	NO	169	+	?	373	−	YES	NS	−	X	YES
Hybrids	Bester	Partially Identifies genitor species: HUS and RUT	700/900	−	NO	169	−	NO	−	−	YES	NS	−	X	YES Maternal species HUS
	Sterbe	Partially identifies genitor species: HUS and RUT	700/900	−	NO	169	−	NO	373	−	NO	NS	−	X	YES Maternal species RUT
	Best beluga	YES	700/700	−	NO	169	−	NO	373	−	NO	NS	−	NO	YES Maternal species HUS
	H203	Partially identifies the possible genitors: HUS, ARU, AGU + ABE	700/900	−	NO	169	−	YES *	373	−	YES **	NS	−	NO	YES Maternal species HUS
	H204	Partially identifies one genitor: AGU/ABE Other possible genitor: ARU/HUS	700/900	−	NO	169/169?	−	YES ***	−	−	YES ****	NS	−	NO	YES Maternal species ARU

* Possible genitors ARU/AGU/HUS; ** possible genitors HUS/ARU, excludes AGU. Final diagnostic: H203 = ♀ HUS *×* ♂ ARU (bester); *** possible genitors ARU/AGU; **** excludes HUS, confirms AGU and ARU; final diagnostic: H204 = ♀ ARU *×* ♂ AGU.

## Data Availability

The data presented in this study are available on request from the corresponding author. The data are not publicly available due to privacy reasons.

## Supplementary Materials

The following supporting information can be downloaded at: https://www.mdpi.com/article/10.3390/foods11071007/s1, Figure S1: Multiple alignment of RP1 locus in *A. ruthenus* and *A. baerii* indicating two putative diagnostic SNPs. Figure S2: Multiple alignment of vimentin locus in *A. stellatus, A. baerii* and *H. huso*. No diagnostic SNPs were detected. Figure S3: Multiple alignment of rhodopsin locus in *A. gueldenstaedtii, A. ruthenus*, *A. stellatus* and *H. huso* indicating two putative diagnostic SNPs.
