# Supplementary material for: A Multistep DNA-Based Methodology for Accurate Authentication of Sturgeon Species
Source: Foods. 2022 Mar 29;11(7):1007. doi: 10.3390/foods11071007 (PMC8998085; doi:10.3390/foods11071007)
Supplement: Supplementary file 1 [file foods-11-01007-s001.zip › foods-1648719-supplementary.pdf]

Supplementary materials

# A Multistep DNA-Based Methodology for Accurate Authentication of Sturgeon Species

**Andreea Dudu [1], Maria Samu [1], Marilena Maereanu [2] and Sergiu Emil Georgescu [1,*]**

[1] Department of Biochemistry and Molecular Biology, Faculty of Biology, University of Bucharest, 050095 Bucharest, Romania; andreea.dudu@bio.unibuc.ro (A.D.); mariasamu95@yahoo.com (M.S.); sergiu.georgescu@bio.unibuc.ro (S.E.G.)

[2] Research Department, SC Danube-Research Consulting, 013172 Isaccea, Romania; marilena.maereanu@gmail.com

* Correspondence: Correspondence: sergiu.georgescu@bio.unibuc.ro; +40-21-3181575/112

```
                            10        20        30        40        50        60        70        80
                   ....|....|....|....|....|....|....|....|....|....|....|....|....|....|....|....|...
Abe76              TTACATTAATTACCTGTGTTACAGATAGATTGTGCCGGGTATTTAGCATTGTGTGTTTCGAGAAAACGTGTCTTGTTTCTTTG
Abe77              TTACATTAATTACCTGTGTTACAGATAGATTGTGCCGGGTATTTAGCATTGTGTGTTTCGAGAAAACGTGTCTTGTTTCTTTG
Abe78              TTACATTAATTACCTGTGTTACAGATAGATTGTGCCGGGTATTTAGCATTGTGTGTTTCGAGAAAACGTGTCTTGTTTCTTTG
Abe79              TTACATTAATTACCTGTGTTACAGATAGATTGTGCCGGGTATTTAGCATTGTGTGTTTCGAGAAAACGTGTCTTGTTTCTTTG
Abe82              TTACATTAATTACCTGTGTTACAGATAGATTGTGCCGGGTATTTAGCATTGTGTGTTTCGAGAAAACGTGTCTTGTTTCTTTG
Aru250             TTACATTAATTACCTGTGTTACAGATAGATTGTGCCGGGTATTTAGCATAGTGTGTTTCGAGAAAACGTGTCTTGTTTCTTTG
Aru251             TTACATTAATTACCTGTGTTACAGATAGATTGTGCCGGGTATTTAGCATAGTGTGTTTCGAGAAAACGTGTCTTGTTTCTTTG
Aru252             TTACATTAATTACCTGTGTTACAGATAGATTGTGCCGGGTATTTAGCATAGTGTGTTTCGAGAAAACGTGTCTTGTTTCTTTG
Aru253             TTACATTAATTACCTGTGTTACAGATAGATTGTGCCGGGTATTTAGCATAGTGTGTTTCGAGAAAACGTGTCTTGTTTCTTTG
Aru254             TTACATTAATTACCTGTGTTACAGATAGATTGTGCCGGGTATTTAGCATAGTGTGTTTCGAGAAAACGTGTCTTGTTTCTTTG
Aru255             TTACATTAATTACCTGTGTTACAGATAGATTGTGCCGGGTATTTAGCATAGTGTGTTTCGAGAAAACGTGTCTTGTTTCTTTG
Clustal Consensus  ************************************************ **********************************

                            110       120       130       140       150       160
                   ....|....|....|....|....|....|....|....|....|....|....|....|....|....
Abe76              ATGTTAAAATCCATATATAGGTAATAAGTTAATAAAATCGGAACTCTTGTTCAAGCTTGTACTTGGATA
Abe77              ATGTTAAAATCCATATATAGGTAATAAGTTAATAAAATCGGAACTCTTGTTCAAGCTTGTACTTGGATA
Abe78              ATGTTAAAATCCATATATAGGTAATAAGTTAATAAAATCGGAACTCTTGTTCAAGCTTGTACTTGGATA
Abe79              ATGTTAAAATCCATATATAGGTAATAAGTTAATAAAATCGGAACTCTTGTTCAAGCTTGTACTTGGATA
Abe82              ATGTTAAAATCCATATATAGGTAATAAGTTAATAAAATCGGAACTCTTGTTCAAGCTTGTACTTGGATA
Aru250             ATGTTAAAATCCATATATAGGTAATAAGTTAATAAAATCGGAACTCGTGTTCAAGCTTGTACTTGGATA
Aru251             ATGTTAAAATCCATATATAGGTAATAAGTTAATAAAATCGGAACTCGTGTTCAAGCTTGTACTTGGATA
Aru252             ATGTTAAAATCCATATATAGGTAATAAGTTAATAAAATCGGAACTCGTGTTCAAGCTTGTACTTGGATA
Aru253             ATGTTAAAATCCATATATAGGTAATAAGTTAATAAAATCGGAACTCGTGTTCAAGCTTGTACTTGGATA
Aru254             ATGTTAAAATCCATATATAGGTAATAAGTTAATAAAATCGGAACTCGTGTTCAAGCTTGTACTTGGATA
Aru255             ATGTTAAAATCCATATATAGGTAATAAGTTAATAAAATCGGAACTCGTGTTCAAGCTTGTACTTGGATA
Clustal Consensus  ********************************************** **********************
```

**Figure S1.** Multiple alignment of RP1 locus in *A. ruthenus* and *A. baerii* indicating two putative diagnostic SNPs.

```
                            10        20        30        40        50        60        70        80
                    ....|....|....|....|....|....|....|....|....|....|....|....|....|....|....|....|...
Abe76_vimentin      TCCAGGGTTTCCTACACCAGCCAATCAGCCCCGACTCTCTACGCGACCAAATCGTCATCCACGAGGCTCAGAAGCAGCGCTCC
Abe77_vimentin      TCCAGGGTTTCCTACACCAGCCAATCAGCCCCGACTCTCTACGCGACCAAATCGTCATCCACGAGGCTCAGAAGCAGCGCTCC
Abe78_vimentin      TCCAGGGTTTCCTACACCAGCCAATCAGCCCCGACTCTCTACGCGACCAAATCGTCATCCACGAGGCTCAGAAGCAGCGCTCC
Abe79_vimentin      TCCAGGGTTTCCTACACCAGCCAATCAGCCCCGACTCTCTACGCGACCAAATCGTCATCCACGAGGCTCAGAAGCAGCGCTCC
Abe82_vimentin      TCCAGGGTTTCCTACACCAGCCAATCAGCCCCGACTCTCTACGCGACCAAATCGTCATCCACGAGGCTCAGAAGCAGCGCTCC
Hh165_vimentin      TCCAGGGTTTCCTACACCAGCCAATCAGCCCCGACTCTCTACGCGACCAAATCGTCATCAACGAGGCTCAGAAGCAGCGCTCC
Hh166_vimentin      TCCAGGGTTTCCTACACCAGCCAATCAGCCCCGACTCTCTACGCGACCAAATCGTCATCCACGAGGCTCAGAAGCAGCGCTCC
Hh167_vimentin      TCCAGGGTTTCCTACACCAGCCAATCAGCCCCGACTCTCTACGCGACCAAATCGTCATCAACGAGGCTCAGAAGCAGCGCTCC
Hh168_vimentin      TCCAGGGTTTCCTACACCAGCCAATCAGCCCCGACTCTCTACGCGACCAAATCGTCATCCACGAGGCTCAGAAGCAGCGCTCC
Hh169_vimentin      TCCAGGGTTTCCTACACCAGCCAATCAGCCCCGACTCTCTACGCGACCAAATCGTCATCAACGAGGCTCAGAAGCAGCGCTCC
Ast154_vimentin     TCCAGGGTTTCCTACACCAGCCAATCAGCCCCGACTCTCTACGCGACCAAATCGTCATCCACGAGGCTCAGAAGCAGCGCTCC
Ast155_vimentin     TCCAGGGTTTCCTACACCAGCCAATCAGCCCCGACTCTCTACGCGACCAAATCGTCATCCACGAGGCTCAGAAGCAGCGCTCC
Ast157_vimentin     TCCAGGGTTTCCTACACCAGCCAATCAGCCCCGACTCTCTACGCGACCAAATCGTCATCCACGAGGCTCAGAAGCAGCGCTCC
Ast158_vimentin     TCCAGGGTTTCCTACACCAGCCAATCAGCCCCGACTCTCTACGCGACCAAATCGTCATCCACGAGGCTCAGAAGCAGCGCTCC
Ast159_vimentin     TCCAGGGTTTCCTACACCAGCCAATCAGCCCCGACTCTCTACGCGACCAAATCGTCATCCACGAGGCTCAGAAGCAGCGCTCC
Clustal Consensus   *********************************************************** ***********************

                           110       120       130       140       150       160       170       180
                    ....|....|....|....|....|....|....|....|....|....|....|....|....|....|....|....|...
Abe76_vimentin      CTGAAACTTTGGATTTCGCCTTGTCCGATGCCATCAACACTGAATTCAAAGCCAACAGAACCAATGAAAAGGCGGAGATGCAG
Abe77_vimentin      CTGAAACTTTGGATTTCGCCTTGTCCGATGCCATCAACACTGAATTCAAAGCCAACAGAACCAATGAAAAGGCGGAGATGCAG
Abe78_vimentin      CTGAAACTTTGGATTTCGCCTTGTCCGATGCCATCAACACTGAATTCAAAGCCAACAGAACCAATGAAAAGGCGGAGATGCAG
Abe79_vimentin      CTGAAACTTTGGATTTCGCCTTGTCCGATGCCATCAACACTGAATTCAAAGCCAACAGAACCAATGAAAAGGCGGAGATGCAG
Abe82_vimentin      CTGAAACTTTGGATTTCGCCTTGTCCGATGCCATCAACACTGAATTCAAAGCCAACAGAACCAATGAAAAGGCGGAGATGCAG
Hh165_vimentin      CTGAAACTTTGGATTTCGCCTTGTCCGATGCCATCAACACTGAATTCAAAGCCAACAGAACCAATGAAAAGGCGGAGATGCAG
Hh166_vimentin      CTGAAACTTTGGATTTCGCCTTGTCCGATGCCATCAACACTGAATTCAAAGCCAACAGAACCAATGAAAAGGCGGAGATGCAG
Hh167_vimentin      CTGAAACTTTGGATTTCGCCTTGTCCGATGCCATCAACACTGAATTCAAAGCCAACAGAACCAATGAAAAGGCGGAGATGCAG
Hh168_vimentin      CTGAAACTTTGGATTTCGCCTTGTCCGATGCCATCAACACTGAATTCAAAGCCAACAGAACCAATGAAAAGGCGGAGATGCAG
Hh169_vimentin      CTGAAACTTTGGATTTCGCCTTGTCCGATGCCATCAACACTGAATTCAAAGCCAACAGAACCAATGAAAAGGCGGAGATGCAG
Ast154_vimentin     CTGAAACTTTGGATTTCGCCTTGTCCGATGCCATCAACACTGAATTCAAAGCCAACAGAACCAATGAAAAGGCGGAGATGCAG
Ast155_vimentin     CTGAAACTTTGGATTTCGCCTTGTCCGATGCCATCAACACTGAATTCAAAGCCAACAGAACCAATGAAAAGGCGGAGATGCAG
Ast157_vimentin     CTGAAACTTTGGATTTCGCCTTGTCCGATGCCATCAACACTGAATTCAAAGCCAACAGAACCAATGAAAAGGCGGAGATGCAG
Ast158_vimentin     CTGAAACTTTGGATTTCGCCTTGTCCGATGCCATCAACACTGAATTCAAAGCCAACAGAACCAATGAAAAGGCGGAGATGCAG
Ast159_vimentin     CTGAAACTTTGGATTTCGCCTTGTCCGATGCCATCAACACTGAATTCAAAGCCAACAGAACCAATGAAAAGGCGGAGATGCAG
Clustal Consensus   ***********************************************************************************

                           210       220       230       240       250       260       270       280
                    ....|....|....|....|....|....|....|....|....|....|....|....|....|....|....|....|...
Abe76_vimentin      TGCCTCCTACATCGATAAGGTGAGGTTTCTGGAGCAGCAGAACAAGATTCTGATGGCCGAGTTGGAGCAGCTGAAGGGAAAAG
Abe77_vimentin      TGCCTCCTACATCGATAAGGTGAGGTTTCTGGAGCAGCAGAACAAGATTCTGATGGCCGAGTTGGAGCAGCTGAAGGGAAAAG
Abe78_vimentin      TGCCTCCTACATCGATAAGGTGAGGTTTCTGGAGCAGCAGAACAAGATTCTGATGGCCGAGTTGGAGCAGCTGAAGGGAAAAG
Abe79_vimentin      TGCCTCCTACATCGATAAGGTGAGGTTTCTGGAGCAGCAGAACAAGATTCTGATGGCCGAGTTGGAGCAGCTGAAGGGAAAAG
Abe82_vimentin      TGCCTCCTACATCGATAAGGTGAGGTTTCTGGAGCAGCAGAACAAGATTCTGATGGCCGAGTTGGAGCAGCTGAAGGGAAAAG
Hh165_vimentin      TGCCTCCTACATCGATAAGGTGAGGTTTCTGGAGCAGCAGAACAAGATTCTGATGGCCGAGTTGGAGCAGCTGAAGGGAAAAG
Hh166_vimentin      TGCCTCCTACATCGATAAGGTGAGGTTTCTGGAGCAGCAGAACAAGATTCTGATGGCCGAGTTGGAGCAGCTGAAGGGAAAAG
Hh167_vimentin      TGCCTCCTACATCGATAAGGTGAGGTTTCTGGAGCAGCAGAACAAGATTCTGATGGCCGAGTTGGAGCAGCTGAAGGGAAAAG
Hh168_vimentin      TGCCTCCTACATCGATAAGGTGAGGTTTCTGGAGCAGCAGAACAAGATTCTGATGGCCGAGTTGGAGCAGCTGAAGGGAAAAG
Hh169_vimentin      TGCCTCCTACATCGATAAGGTGAGGTTTCTGGAGCAGCAGAACAAGATTCTGATGGCCGAGTTGGAGCAGCTGAAGGGAAAAG
Ast154_vimentin     TGCCTCCTACATCGATAAGGTGAGGTTTCTGGAGCAGCAGAACAAGATTCTGATGGCCGAGTTGGAGCAGCTGAAGGGAAAAG
Ast155_vimentin     TGCCTCCTACATCGATAAGGTGAGGTTTCTGGAGCAGCAGAACAAGATTCTGATGGCCGAGTTGGAGCAGCTGAAGGGAAAAG
Ast157_vimentin     TGCCTCCTACATCGATAAGGTGAGGTTTCTGGAGCAGCAGAACAAGATTCTGATGGCCGAGTTGGAGCAGCTGAAGGGAAAAG
Ast158_vimentin     TGCCTCCTACATCGATAAGGTGAGGTTTCTGGAGCAGCAGAACAAGATTCTGATGGCCGAGTTGGAGCAGCTGAAGGGAAAAG
Ast159_vimentin     TGCCTCCTACATCGATAAGGTGAGGTTTCTGGAGCAGCAGAACAAGATTCTGATGGCCGAGTTGGAGCAGCTGAAGGGAAAAG
Clustal Consensus   ***********************************************************************************

                           310       320       330       340       350       360       370
                    ....|....|....|....|....|....|....|....|....|....|....|....|....|....|...
Abe76_vimentin      GACCTCTATGAGGACGAGATGAGGGAGCTCCGTCGTCAGGTGGACAAGCAAACCAACGAAAAGGCGAGGGTGG
Abe77_vimentin      GACCTCTATGAGGACGAGATGAGGGAGCTCCGTCGTCAGGTGGACAAGCAAACCAACGAAAAGGCGAGGGTGG
Abe78_vimentin      GACCTCTATGAGGACGAGATGAGGGAGCTCCGTCGTCAGGTGGACAAGCAAACCAACGAAAAGGCGAGGGTGG
Abe79_vimentin      GACCTCTATGAGGACGAGATGAGGGAGCTCCGTCGTCAGGTGGACAAGCAAACCAACGAAAAGGCGAGGGTGG
Abe82_vimentin      GACCTCTATGAGGACGAGATGAGGGAGCTCCGTCGTCAGGTGGACAAGCAAACCAACGAAAAGGCGAGGGTGG
Hh165_vimentin      GACCTCTATGAGGACGAGATGAGGGAGCTCCGTCGTCAGGTGGACAAGCAAACCAACGAAAAGGCGAGGGTGG
Hh166_vimentin      GACCTCTATGAGGACGAGATGAGGGAGCTCCGTCGTCAGGTGGACAAGCAAACCAACGAAAAGGCGAGGGTGG
Hh167_vimentin      GACCTCTATGAGGACGAGATGAGGGAGCTCCGTCGTCAGGTGGACAAGCAAACCAACGAAAAGGCGAGGGTGG
Hh168_vimentin      GACCTCTATGAGGACGAGATGAGGGAGCTCCGTCGTCAGGTGGACAAGCAAACCAACGAAAAGGCGAGGGTGG
Hh169_vimentin      GACCTCTATGAGGACGAGATGAGGGAGCTCCGTCGTCAGGTGGACAAGCAAACCAACGAAAAGGCGAGGGTGG
Ast154_vimentin     GACCTCTATGAGGACGAGATGAGGGAGCTCCGTCGTCAGGTGGACAAGCAAACCAACGAAAAGGCGAGGGTGG
Ast155_vimentin     GACCTCTATGAGGACGAGATGAGGGAGCTCCGTCGTCAGGTGGACAAGCAAACCAACGAAAAGGCGAGGGTGG
Ast157_vimentin     GACCTCTATGAGGACGAGATGAGGGAGCTCCGTCGTCAGGTGGACAAGCAAACCAACGAAAAGGCGAGGGTGG
Ast158_vimentin     GACCTCTATGAGGACGAGATGAGGGAGCTCCGTCGTCAGGTGGACAAGCAAACCAACGAAAAGGCGAGGGTGG
Ast159_vimentin     GACCTCTATGAGGACGAGATGAGGGAGCTCCGTCGTCAGGTGGACAAGCAAACCAACGAAAAGGCGAGGGTGG
Clustal Consensus   *************************************************************************
```

**Figure S2.** Multiple alignment of vimentin locus in *A. stellatus, A. baerii* and *H. huso*. No diagnostic SNPs were detected.

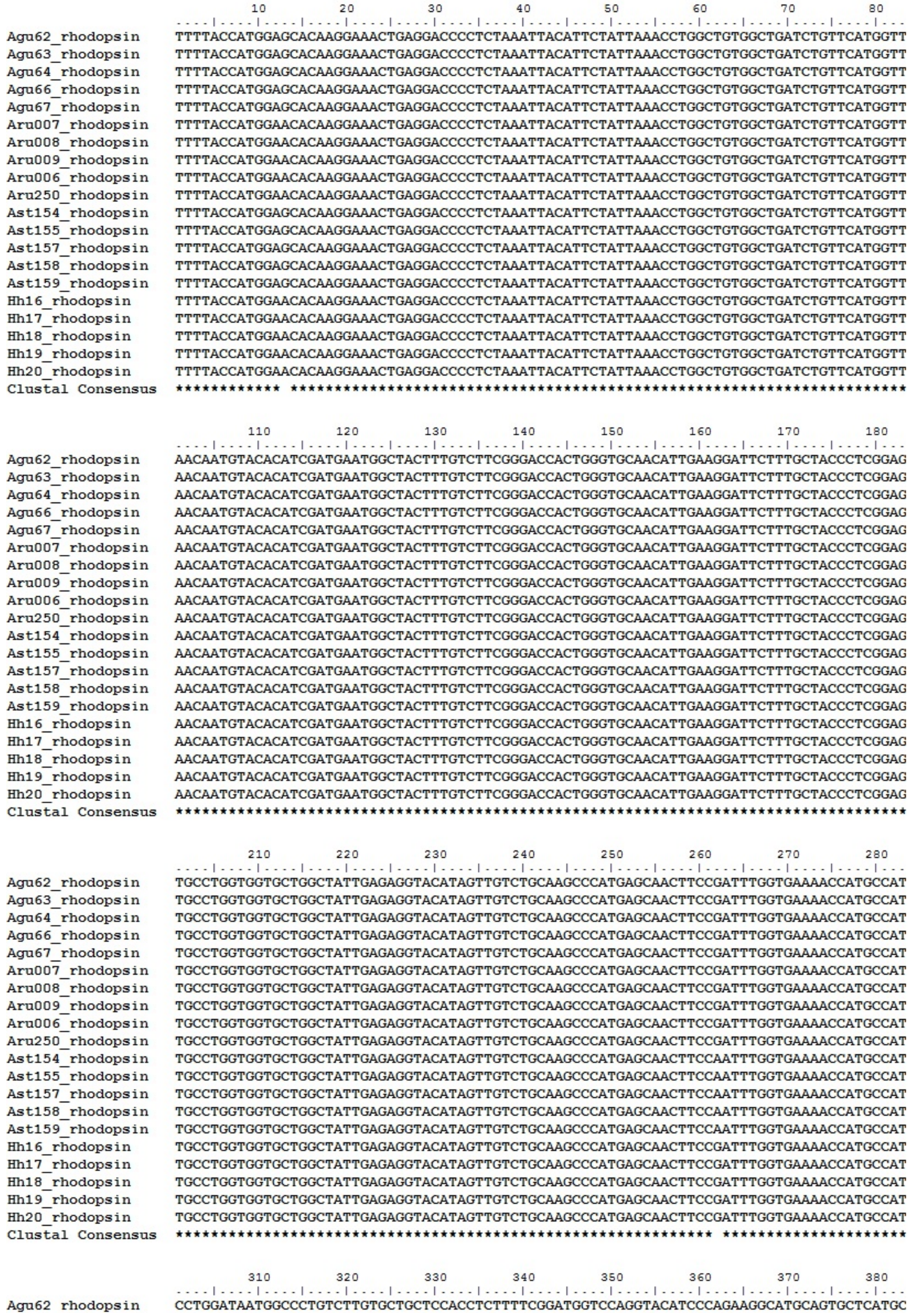


**Figure S3.** Multiple alignment of rhodopsin locus in *A. gueldenstaedtii, A. ruthenus, A. stellatus* and *H. huso* indicating two putative diagnostic SNPs.
